# Skin Hyperpigmentation as Initial Manifestation of Vitamin B_12_ Deficiency: A Case of Delayed Diagnosis With Late‐Onset Subacute Combined Degeneration of the Cord

**DOI:** 10.1155/carm/4459475

**Published:** 2026-02-11

**Authors:** Prosper Adjei, Kingsley Owusu Manu, Paa Kwesi Asante Addison, Emmanuel Ntow

**Affiliations:** ^1^ Department of Internal Medicine, Methodist Hospital, Wenchi, Ghana

**Keywords:** ataxic gait, cobalamin deficiency, hyperpigmentation, macrocytic anemia, subacute combined degeneration of the cord, vitamin B_12_ deficiency

## Abstract

Vitamin B_12_ deficiency may present with a myriad of symptoms. Rarely, skin hyperpigmentation may be the sole initial manifestation of this micronutrient deficiency, which can easily be overlooked, leading to delayed diagnosis. This case report describes a 32‐year‐old Ghanaian male who presented with a 4‐year history of progressive darkening of the dorsal aspects of the interphalangeal joints of both hands, the palms, and soles of the feet, for which he did not seek immediate medical attention. He later developed a worsening unsteady gait. Neurological assessment revealed mild weakness in both lower limbs, brisk knee reflexes, diminished ankle reflexes, and bilateral extensor plantar reflexes. Additionally, he had decreased sensitivity to fine touch, impaired proprioception, and diminished vibration sense. His gait was ataxic with a positive Romberg’s sign. Laboratory investigations showed macrocytosis with anemia and a markedly reduced serum vitamin B_12_ level. Upper gastrointestinal endoscopy with biopsy identified chronic gastritis. He was diagnosed with vitamin B_12_ deficiency secondary to chronic gastritis complicated by subacute combined degeneration of the cord. Following parenteral repletion of vitamin B_12_, the skin hyperpigmentation completely resolved. His neurological symptoms also significantly improved. This case report highlights the need to recognize skin hyperpigmentation as a potential initial symptom of cobalamin deficiency, which is critical for early diagnosis and prompt initiation of treatment to prevent debilitating neurological complications.

## 1. Introduction

Vitamin B_12_, also known as cobalamin, is a water‐soluble vitamin obtained mainly from animal‐based foods such as fish, meat, poultry, eggs, milk, and other dairy products [1]. It is an essential micronutrient required for hematopoiesis and normal neuronal function [2]. It plays a vital role in myelination and function of the central nervous system, as well as deoxyribonucleic acid (DNA) synthesis [3].

On average, the body stores a total of 2–5 mg of vitamin B_12_, primarily in the liver [4]. This far exceeds the recommended daily amount of 2.4 μg for adults [3]. Cobalamin is highly conserved through the enterohepatic circulation, and therefore, deficiency resulting from malabsorption typically occurs after 2–5 years. Due to the large reserve of vitamin B_12_ in humans, symptoms arising from inadequate dietary intake can take several years (usually after 10–20 years) to appear [4].

Although cobalamin deficiency poses a significant health challenge globally, the prevalence often tends to be higher in low‐ and middle‐income countries [5]. In Ghana, however, there is a paucity of data on the burden of cobalamin deficiency. One national survey estimated the prevalence of vitamin B_12_ deficiency among nonpregnant women to be 6.9% [6], a relatively low rate compared to other micronutrient deficiencies in Ghana.

There are several etiological factors for vitamin B_12_ deficiency. Reduced dietary intake (strict vegetarian or vegan diet), pernicious anemia, chronic gastritis, gastrectomy, and ileal resection are recognized causes of this micronutrient deficiency. Other conditions like Crohn’s disease, small intestinal bacterial overgrowth, and pancreatic insufficiency have also been implicated [7]. Additionally, long‐term use of medications such as metformin, proton pump inhibitors, and histamine‐2 receptor antagonists can result in vitamin B_12_ deficiency. *Dibothriocephalus latus* can cause low vitamin B_12_ levels through dissociation of the vitamin B_12_‐intrinsic factor complex and via competition with the human host for vitamin B_12_ absorption in the gut [7].

Individuals with vitamin B_12_ deficiency may have hematologic, neuropsychiatric, dermatologic, and cardiovascular manifestations [4]. The diverse and nonspecific nature of these clinical features can make the diagnosis of vitamin B_12_ deficiency quite challenging, particularly in resource‐constrained settings. Rarely, skin hyperpigmentation may be the sole initial manifestation of cobalamin deficiency [8], which can easily be overlooked, especially in individuals with dark skin tones, leading to delayed diagnosis and potentially severe complications. This case report describes a young Ghanaian male with vitamin B_12_ deficiency, manifesting initially as skin hyperpigmentation, which was overlooked and later was complicated by subacute combined degeneration of the cord.

## 2. Case Presentation

A 32‐year‐old Ghanaian male presented to our hospital with a 4‐year history of progressive darkening of both hands and feet. The darkening of the skin started on the dorsal aspects of the proximal and distal interphalangeal joints of both hands and slowly progressed to the palms and soles of the feet. It was not initially associated with any systemic symptoms, and there was no antecedent history of skin rash or allergic drug reaction. He reported that the darkening of his skin was not bothersome as he naturally had a dark skin tone for which reason he did not seek immediate medical attention. About 2 years after the onset of the aforementioned symptom, he had recurrent fatigue and generalized weakness which prompted him to pursue medical care at different health facilities. He was found to be severely anemic on three different occasions and was accordingly transfused. However, he did not know whether any specific diagnostic tests for the cause of the recurrent anemia were performed at the various health facilities he visited earlier. Four months prior to presentation, he developed an unsteady gait which started insidiously and gradually worsened to the point where he could not walk without support. The unsteady gait was preceded by paresthesia and tingling sensation of his feet and was particularly aggravated in dark environments. There was, however, no urinary or fecal incontinence. Also, there was no hallucination, impaired memory, or abnormal behavior. He had no previous history of gastrointestinal surgery, malabsorption disorders, or any chronic diseases. He was not on any long‐term medications and did not abuse nonsteroidal anti‐inflammatory drugs. There was no significant history of familial illness. He was neither a vegan nor a vegetarian and equally did not drink alcohol or smoke cigarettes.

**FIGURE 1:**
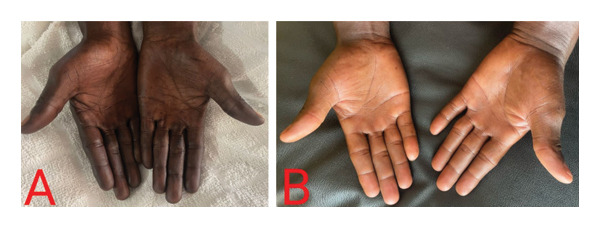
(A) Hyperpigmentation of both palms of the patient at initial presentation. (B) Complete resolution of palmar hyperpigmentation 2 months after starting parenteral cyanocobalamin.

**FIGURE 2:**
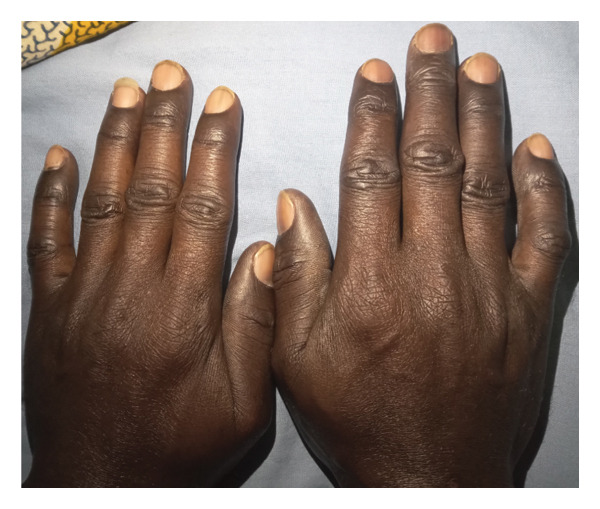
Hyperpigmentation of the dorsal aspects of the proximal and distal interphalangeal joints.

**FIGURE 3:**
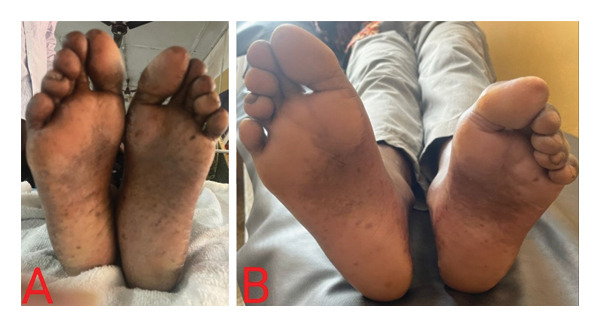
(A) Hyperpigmentation of the soles of the feet at initial presentation. (B) Reversal of hyperpigmentation of the soles of the feet 2 months after initiating parenteral cyanocobalamin.

On physical assessment, he had a normal weight with a body mass index of 23.5 kg/m^2^. He was afebrile (36.3°C), anicteric, moderately pale, and not dyspneic. He had a beefy red tongue but no angular stomatitis. There was hyperpigmentation of the palms (Figure 1(A)), dorsal aspects of the proximal and distal interphalangeal joints of both hands (Figure 2), and the soles of the feet (Figure 3(A)). His nails, hair, and mucosal surfaces were normal. Neurological examination of the lower limbs showed mildly reduced muscle power (Medical Research Council Grade 4) with increased tone. In addition, knee reflexes were brisk while ankle reflexes were diminished. There was bilateral extensor plantar response. Sensory evaluation revealed decreased sensitivity to fine touch (hypoesthesia), impaired proprioception, and diminished vibration sense (elicited by using a 128‐Hz tuning fork). However, temperature and pain sensation remained intact. His gait was ataxic with a positive Romberg’s sign and inability to perform a straight‐line tandem walk. The cranial nerve examination and ophthalmological assessment were unremarkable. Neurological evaluation of the upper limbs and examination of the other systems were normal.

**FIGURE 4:**
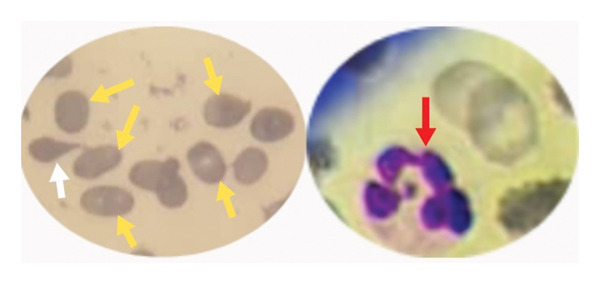
Peripheral blood smear showing macro‐ovalocytes (yellow arrows), tear drop cell (white arrow), and hypersegmented neutrophil (red arrow).

**FIGURE 5:**
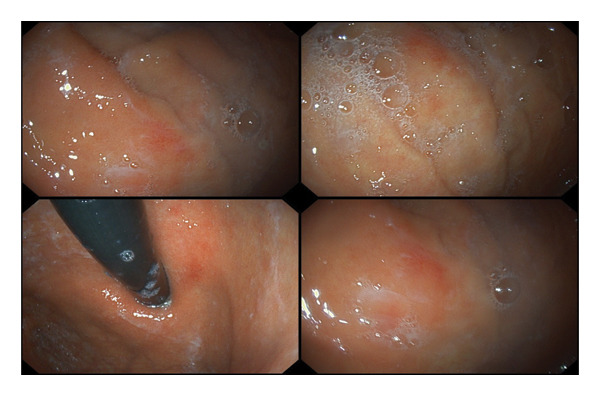
Endoscopic images of the patient demonstrating gastric mucosal erythema.

Initial laboratory investigations revealed severe macrocytic anemia (hemoglobin = 6.5 g/dL; mean corpuscular volume = 105.3 fL) with pancytopenia (white blood cell = 1.66 × 10^3^/μL; platelet = 67.0 × 10^9^/L). Notable findings on peripheral blood smear were marked macro‐ovalocytosis, hypersegmented neutrophils, and anisopoikilocytosis (Figure 4). The reticulocyte count was also low. To identify the cause of macrocytic anemia and neurological features in this patient, serum vitamin B_12_ and folate tests were conducted, which showed a significantly reduced vitamin B_12_ level (37 pmol/L) and an elevated serum folate level (38.1 nmol/L). Indirect bilirubin, lactate dehydrogenase and renal biochemistries were within normal range. Anti‐intrinsic factor and antiparietal cell antibodies, in addition to serological screening tests, were negative. Stool microscopic examination was unremarkable. Table 1 captures the results of all relevant laboratory tests for the patient. Magnetic resonance imaging (MRI) of the cervicothoracic spine was not performed due to financial constraints. Further investigations to unravel the cause of vitamin B_12_ deficiency included upper gastrointestinal endoscopy, which demonstrated mucosal erythema in the fundus and body of the stomach (Figure 5). Histopathological examination of endoscopic biopsies showed increased chronic inflammatory infiltrates in the lamina propria, consistent with chronic gastritis. Subsequent *Helicobacter pylori* stool antigen test was negative.

**TABLE 1:** Results of laboratory tests for the patient.

Laboratory parameter (unit)	Initial result	Result at 2‐month follow‐up	Reference range
White blood cell (× 10^3^/μL)	1.66	5.8	4.00–10.00
Red blood cell (× 10^6^/μL)	1.71	5.78	4.50–6.50
Hemoglobin (g/dL)	6.5	15.6	11.00–16.00
MCV (fL)	105.3	86.0	80.00–100.00
MCH (pg)	29.0	28.7	26–33
Platelet (× 10^9^/L)	67.0	276	150.00–450.00
Reticulocyte count (%)	0.3	1.5	0.5–2.0
Serum vitamin B_12_ (pmol/L)	37	265	133–675
Serum folate (nmol/L)	38.1	14.6	6.0–28.0
Intrinsic factor antibodies	Negative		
Parietal cell antibodies	Negative		
Indirect bilirubin (μmol/L)	10.8		1.7–17
LDH (U/L)	208		135–225
Urea (mmol/L)	3.2		2.0–8.3
Creatinine (μmol/L)	95		55.0–110.0
HIV	Negative		
VDRL	Negative		
HBsAg	Negative		
HCV antibodies	Negative		
Stool microscopy	No parasites or ova seen		
*H. pylori* stool antigen	Negative		

*Note:* LDH, lactate dehydrogenase; HBsAg, hepatitis B surface antigen.

Abbreviations: HCV, hepatitis C virus; HIV, human immunodeficiency virus; MCH, mean corpuscular hemoglobin; MCV, mean corpuscular volume; VDRL, venereal disease research laboratory.

Based on the above, a diagnosis of vitamin B_12_ deficiency secondary to chronic gastritis complicated by subacute combined degeneration of the cord was made. Intramuscular cyanocobalamin was started at 1000 μg daily for 7 days, then weekly for 4 weeks, and then monthly thereafter. Additionally, he received oral omeprazole 20 mg twice daily for 14 days. Physiotherapy was initiated to address the mobility and functional limitations. Nutritional counseling was equally provided, and he was advised to increase dietary intake of vitamin B_12._

At follow‐up 2 months after the initiation of intramuscular cyanocobalamin, the macrocytic anemia and pancytopenia had completely resolved. Also, serum vitamin B_12_ and folate levels had normalized (Table 1). The paresthesia and tingling sensation had resolved with significantly improved ataxic gait such that he could walk without support and was able to do his daily activities without any struggle. Again, there was complete reversal of the skin hyperpigmentation (Figures 1(B) and 3(B)).

## 3. Discussion

Patients with vitamin B_12_ deficiency may be asymptomatic or have a wide range of symptoms. Rarely, hyperpigmentation can be an early clinical feature or the only manifestation of vitamin B_12_ deficiency [8–10]. Hyperpigmentation associated with cobalamin deficiency is often encountered in dark‐skinned people [11]. About 1 in 5 persons deficient in cobalamin may have this particular dermatological manifestation. The exact pathophysiologic mechanism is not fully understood. It has been suggested that hyperpigmentation is caused by decreased level of reduced glutathione, which enhances tyrosinase activity and leads to increased melanin production. A defect in melanin transfer from melanocytes to keratinocytes is also thought to play a role in hyperpigmentation of the skin. It typically affects the dorsal aspects of the hands and feet, palms, soles, nails, and oral mucosa [8, 9]. Other notable mucocutaneous features of cobalamin deficiency are vitiligo, hair changes (graying and textural changes), nail changes, glossitis, and angular stomatitis [8, 9, 11].

As noted from the patient’s clinical history, skin hyperpigmentation occurred several months before the onset of other systemic symptoms. In individuals with dark skin tones, hyperpigmentation of the aforementioned areas can be easily overlooked, especially if it is the only manifestation of vitamin B_12_ deficiency. This may lead to delayed diagnosis, which can culminate in neurological complications, as was observed in this patient. In a similar case reported in Ghana, a 33‐year‐old male presented with progressive hyperpigmentation of his palms and feet, which he initially attributed to occupational exposures, resulting in a delay in seeking medical care. He later developed severe macrocytic anemia but had no neurological signs. He was eventually diagnosed with vitamin B_12_ deficiency secondary to *Helicobacter pylori*–associated gastritis after an extensive diagnostic work‐up [10]. It is imperative for clinicians to consider vitamin B_12_ deficiency as a differential diagnosis when evaluating patients with skin hyperpigmentation.

Although the patient received multiple blood transfusions at various health facilities on account of recurrent symptomatic anemia, it is uncertain whether any specific diagnostic tests were carried out to determine the cause of the anemia. This missed opportunity contributed greatly to the delayed diagnosis of the micronutrient deficiency as well as the subsequent development of neurological complications in the index patient. At presentation, his initial complete blood count showed severe macrocytic anemia with pancytopenia. Also, the peripheral blood smear classically demonstrated macro‐ovalocytes and hypersegmented neutrophils, which are pathognomonic findings in megaloblastic anemia [12]. Macrocytic anemia is the commonest hematological abnormality associated with cobalamin deficiency. Decreased levels of cobalamin cause ineffective erythropoiesis by interfering with DNA synthesis in hematopoietic cells. This leads to the formation of abnormally large, immature red blood cells [12]. Pancytopenia, as seen in our patient, is a rare hematological complication of cobalamin deficiency, which is encountered in only 5% of affected individuals [7, 13]. It must be emphasized that normal mean corpuscular volume does not rule out cobalamin deficiency, and so serum vitamin B_12_ levels should be measured whenever a deficient state is strongly suspected [2].

People with cobalamin deficiency can have a myriad of neurological manifestations, which include peripheral neuropathy, myelopathy, cognitive impairment, and optic neuropathy [4, 14]. In a previously published case report by Yakubu et al., a patient with cobalamin deficiency presented with skin hyperpigmentation, paresthesia and ataxic gait. Unlike the index case, the patient described by Yakubu et al. also had psychiatric symptoms [2]. Meticulous neurological assessment of our patient revealed findings suggestive of subacute combined degeneration of the cord. This is an uncommon neurological sequela of cobalamin deficiency characterized by demyelination of the dorsal and lateral columns of the spinal cord [15]. Vitamin B_12_ serves as a cofactor for methionine synthase and methylmalonyl‐coA mutase. Methionine synthase converts homocysteine to methionine, which is then used to produce S‐adenosylmethionine, an important methyl donor required for maintaining myelin sheath integrity. Vitamin B_12_ deficiency decreases production of S‐adenosylmethionine, impairing the methylation of myelin proteins and lipids which eventually results in myelin sheath damage [16]. In addition, methylmalonyl‐coA mutase converts methylmalonyl‐coA to succinyl‐CoA, a compound used in the formation of myelin. Depletion of vitamin B_12_ levels, thus hinders the normal synthesis of myelin [16].

Subacute combined degeneration frequently affects the cervical and thoracic regions of the spinal cord. MRI of the spine characteristically shows bilateral symmetric T2 hyperintense signal in the dorsal columns. This radiological finding is mostly referred to as the “inverted V” or “inverted rabbit ears” sign [16, 17]. MRI was not performed for our patient because he was financially constrained. As a result, the diagnosis of subacute combined degeneration of the cord was based on the combination of neurological signs elicited during physical examination and confirmed vitamin B_12_ deficiency. Additionally, the patient’s positive response to vitamin B_12_ replacement therapy lent further credence to the diagnosis of this neurological complication. Although MRI is a valuable diagnostic tool, it may be normal in 60%–85% of patients with subacute combined degeneration of the cord [17].

Serum vitamin B_12_ and folate levels are required in the evaluation of patients with suspected vitamin B_12_ deficiency. In instances where these tests are unrevealing, methylmalonic acid and homocysteine levels can be measured [18]. Our patient’s initial laboratory tests demonstrated extremely low serum vitamin B_12_ level and elevated folate level (Table 1). Inadequate vitamin B_12_ decreases the activity of methionine synthase. This results in the accumulation of 5‐methyltetrahydrofolate, causing a spuriously high folate level [19] as noted in the index case. The underlying etiology of vitamin B_12_ deficiency was not apparent from the patient’s clinical history and initial laboratory investigations. This necessitated further evaluation with upper gastrointestinal endoscopy and histopathological examination of gastric biopsies, which led to the discovery of features suggestive of chronic gastritis. Destruction of parietal cells by chronic gastritis leads to reduced secretion of hydrochloric acid and intrinsic factor, which results in malabsorption of vitamin B_12_ [10, 20].

Although Addison disease, hyperthyroidism, and hemochromatosis are frequently associated with hyperpigmentation, the coexistence of macrocytic anemia and subacute combined degeneration of the cord made these conditions unlikely for which reason they were not considered in our patient.

Repletion of the deficient vitamin is the mainstay of treatment for cobalamin deficiency. Vitamin B_12_ is administered either parenterally or orally depending on the underlying cause and the severity of symptoms [18, 21]. In the case of our patient, cyanocobalamin was administered intramuscularly to prevent irreversible neurological damage and also to correct severe macrocytic anemia (i.e., hemoglobin of 6.5 g/dL).

## 4. Limitation

An objective mental status assessment of the patient using a standardized tool was not performed.

## 5. Conclusion

Rarely, hyperpigmentation can be an early manifestation or the only presenting symptom of vitamin B_12_ deficiency. In dark‐skinned individuals, it may easily be overlooked, leading to delayed diagnosis. It is important for clinicians to consider this micronutrient deficiency as a differential diagnosis when evaluating patients with hyperpigmentation. In countries like Ghana where nutritional deficiencies are prevalent, it may be necessary for clinicians to screen for vitamin B_12_ deficiency in patients presenting with unexplained skin hyperpigmentation. Early recognition and prompt initiation of treatment are crucial to prevent the occurrence of disabling neurological complications.

## Author Contributions

Prosper Adjei: conceptualization and data curation; investigation; writing–original draft; and writing–review and editing. Kingsley Owusu Manu: data curation. Paa Kwesi Asante Addison: data curation. Emmanuel Ntow: data curation.

## Funding

The authors received no financial support for the authorship and/or publication of this article.

## Disclosure

All the authors have read and approved the final manuscript for publication.

## Ethics Statement

Our institution does not require ethical approval for reporting individual cases or case series.

## Consent

Written informed consent was obtained from the patient to publish this article.

## Conflicts of Interest

The authors declare no conflicts of interest.

## Data Availability

Data sharing is not applicable as no datasets were generated or analyzed in this current study.
